# Serum levels of VEGF and MCSF in HER2+ / HER2- breast cancer patients with metronomic neoadjuvant chemotherapy

**DOI:** 10.1186/s40364-018-0135-x

**Published:** 2018-06-14

**Authors:** Roberto J. Arai, Vanessa Petry, Paulo M. Hoff, Max S. Mano

**Affiliations:** Departamento de Radiologia e Oncologia, Instituto do Câncer do Estado de São Paulo, Hospital das Clínicas da Faculdade de Medicina da Universidade de São Paulo, CEP 01246-000, Av. Dr. Arnaldo, São Paulo, SP 251 Brazil

**Keywords:** Metronomic chemotherapy, Angiogenesis, Biomarker, Neoadjuvant, Breast cancer

## Abstract

Metronomic therapy has been gaining importance in the neoadjuvant setting of breast cancer treatment. Its clinical benefits may involve antiangiogenic machinery. Cancer cells induce angiogenesis to support tumor growth by secreting factors, such as vascular endothelial growth factor (VEGF). In breast cancer, Trastuzumab (TZM) based treatment is of key importance and is believed to reduce diameter and volume of blood vessels as well as vascular permeability. Here in we investigated serum levels of angiogenic factors VEGF and MCSF in patients receiving metronomic neoadjuvant therapy with or without TZM. We observed in HER2+ cohort stable levels of MCSF through treatment, whereas VEGF trend was of decreasing levels. In HER2- cohort we observed increasing levels of MCSF and VEGF trend. Overall, HER2+ patients had better pathological response to treatment. These findings suggest that angiogenic pathway may be involved in TZM anti-tumoral effect in the neoadjuvant setting.

## Background

Neoadjuvant chemotherapy was initially indicated to convert a nonresectable into a resectable lesion [1, 2]. Data on the efficacy and safety of metronomic chemotherapy in the neoadjuvant setting for breast cancer (BC) is accumulating and supporting application [3–6]. A down staging of operable tumors, allowing for breast-conserving surgery, is also a potential benefit. It is reported that the average response rates - complete response (CR) plus partial response (PR) - and overall clinical benefit (CR + PR + stable disease [SD] > 6 months) of metronomic chemotherapy in BC management reaches 39% (range 12–88%) and 57% (range 24–93%), respectively [7]. The underlying molecular mechanism of clinical benefit, however, still needs clarification. The concept of a treatment that is delivered more frequently with no prolonged drug-free breaks and with low doses has been proven to target tumor angiogenesis, shifting the tumor vasculature [5, 8]. It may increase the antiangiogenic properties of chemotherapeutic drugs in vitro and in vivo studies [9] including rectal carcinomas [10]. Proliferation and/or induction of apoptosis of activated endothelial cells (ECs) is selectively inhibited as well as inhibition of migration of EC, increase in the expression of thrombospondin-1 and sustained decrease in levels and viability of bone marrow-derived endothelial progenitor cells [11, 12]. Endothelial toxicity caused by anticancer treatment is not considered an antiangiogenic activity but rather alterations of mechanistic regulators including IL-1 and 6, VEGF, VEGFR1 and 2, bFGF, Ang 1 and 2 and MMP-2 [13]. We previously demonstrated that a neoadjuvant metronomic chemotherapy is feasible in two cohorts of HER2+ and HER2− locally advanced BC patients. The complete pathological response (pCR) rates were 55 and 18% among patients enrolled in HER2+ and HER2− cohorts, respectively [14]. In the seminal study conducted by Bottini and coworkers, the overall response rate was 71.9% in the 57 patients randomly assigned to receive primary letrozole and 87.7% in the 57 patients randomly assigned to receive metronomic letrozole plus cyclophosphamide, respectively. In this study, adverse events were comparable between arms. A significantly greater suppression of Ki-67 and VEGF-A expression in the letrozole and cyclophosphamide-treated group compared to the letrozole-only group was found, with a consequent lower Ki-67 and VEGF expression at post-treatment residual histology [3].

TZM is believed to reduce diameter and volume of blood vessels as well as vascular permeability. Studies addressing factors associated with angiogenesis demonstrated that TZM resulted in downregulation of mRNA of the VEGF, transforming growth factor α, plasminogen activator inhibitor 1 and angiopoietin 1 while upregulating antiangiogenic factors such as thrombospondin 1 [15]. The mechanism by which TZM coordinates its antitumor activity through angiogenesis pathway is, however, not well understood and need investigations. An important pathway may include signaling factor VEGF. It is suggested as important mediator for transition of pre-invasive to invasive BC [16]. VEGF expression is in turn regulated by MCSF through monocytes to produce biologically active angiogenic factors [17]. Drugs that target VEGF and VEGF receptor made a major impact, however, we have difficulties in identifying which patient would benefit from then [18–20]. A biomarker to predict which patient would benefit from experiencing the most activity and least toxicity would be of scientific relevance. The objective of the present work is to investigate a possible association of angiogenesis machinery of metronomic chemotherapy and TZM treatment in the neoadjuvant setting. For this purpose, we assessed the plasma levels of VEGF and MCSF in BC patients treated in a clinical trial with metronomic neoadjuvant therapy with or without a TZM containing regimen.

## Methods

### Study design, participants and sample collection

Eligible patients required histological confirmation of invasive ductal carcinoma by core biopsy. Patients diagnosed with inflammatory BC diagnosis were allowed to enter the study. Patients diagnosed with HER2+ BC, defined by HER2 overexpression or amplification, as defined by the American Society of Clinical Oncology/College of American Pathologist (ASCO/CAP), 2007 HER2 testing guidelines – which was the most widely accepted international guidance by the time the study started [21].

All patients received weekly paclitaxel at 100 mg/m^2^ for 8 weeks, followed by weekly doxorubicin at 24 mg/m^2^ for 9 weeks in combination with oral cyclophosphamide at daily dose of 100 mg. HER2+ group received at loading dose of 4 mg/kg TZM followed by a maintenance dose of 2 mg/kg during entire chemotherapy treatment. The total duration of neoadjuvant therapy was 17 weeks. Serum samples were collected from patients at the Instituto do Cancer do Estado de São Paulo (ICESP) included in the clinical research (NCT01329640 and NCT01329627). Prospective sample collections in this phase II study were performed at baseline (±7 days) from first dose and at each 3 weeks (± 7 days) until week 18. Samples were collected according to institutional Standard Operational Procedures. Briefly, plasma samples were collected in EDTA containing tubes. Samples were incubated 30 min at room temperature and centrifuged at 1500 g for 10 min at 4 °C. Aliquots were then frozen at -80 °C until test. Unviable samples such as those with hemolysis were excluded from analysis [22]. Plasma levels of VEGF and MCSF were analyzed by ELISA method according to manufactures’ protocol. The ELISA kits were purchased from R & D Systems.

### Statistical analysis

The chosen method of handling the missing data was the Last Observation Carried Forward (LOCF) [23] as to maintain patient individual response. After run tests we defined that the missing values were not at random. Samples were grouped in weeks (1 ± 1 at week 1, 4, 7, 10 13 and 16). Two patients of the HER2- group were excluded from analysis because number of samples collection was < 50% for each patient. The level of VEGF and MCSF were measured in samples from HER2+ (*n* = 96) and HER2- (*n* = 108) patients. Data were analyzed by using linear regression trend line. Descriptive statistics was used to summarize the results.

### Ethical considerations and standards for human research

This work has been carried out in accordance with The Code of Ethics of the World Medical Association, Declaration of Helsinki and Good Clinical Practice. The informed consent form was obtained accordingly to current local and international legislation and standards. The privacy rights were granted.

## Patients and results

Twenty patients consented to participate and authorized blood collection. Nine patients expressed HER2 (HER2+) and 11 were considered HER2 negative (HER2-). Thirty six percent of patients from HER2- and all patients from HER2+ had stage III BC. Of note, 33% of HER2+ and 66% of HER2- displayed hormone receptor positivity in tumor tissue (Table 1). HER2+ patients receiving TZM presented a pCR rate of 55,5% vs 18% for HER2- patients (Table 2).

**Table 1:** Enrolled patients characteristics

Study group		Patients
Breast cancer	Adenocarcinoma	20
		47,5
Median age (range)		(32–69)
Clinical status	IIA	1
	IIB	3
	IIIA	12
	IIIB	3
	IIIC	1
Histologic grade	1	1
	2	8
	3	11
HER2 status	positive	9
	negative	11
ER status	positive	11
	negative	8
	unknown	1
PR status	positive	13
	negative	7

**Table 2:** Response to treatment

Group (n)	Treatment	Complete pathological response (pCR)	Outcome description
HER2+ [9]	Paclitaxel Doxorubicin Cyclophosphamide and TZM	55,5%	Five patients were alive with no evidence of disease recurrence; Three patients had disease recurrence; One patient deceased with disease progression (median follow-up 33.6 months)
HER2- [11]	Paclitaxel Doxorubicin Cyclophosphamide	18%	Four patients had systemic disease recurrence; one patient deceases with disease progression; two patients were under palliative care; seven patients lived with no evidence of disease recurrence (median follow-up 36.1 months)

### VEGF and MCSF determination

**Fig. 1:**
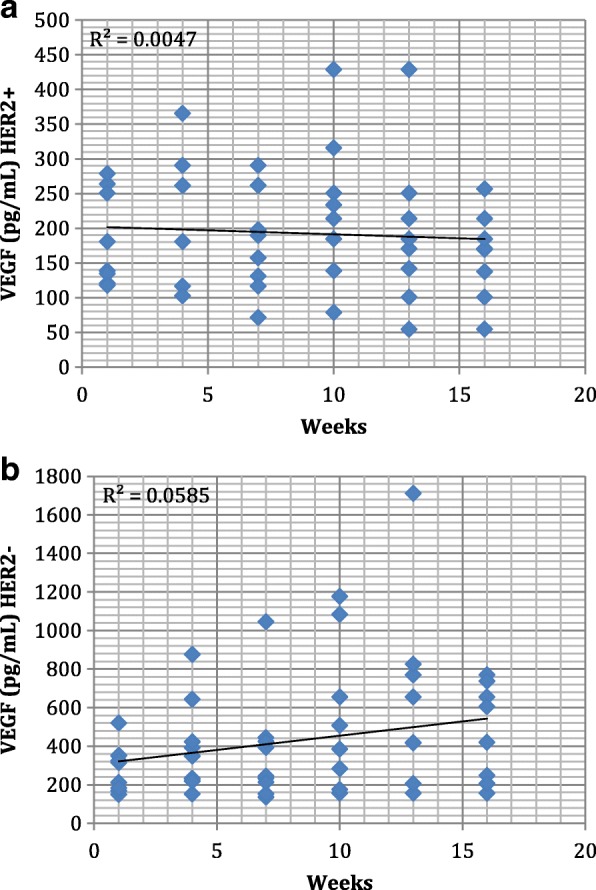
Table (**a**) Two-way scatter plots of VEGF serum levels (*n* = 96) in HER2+ patients and (**b**) HER2- patients (*n* = 108). Trend line along with R^2^ values is shown for each graph

**Fig. 2:**
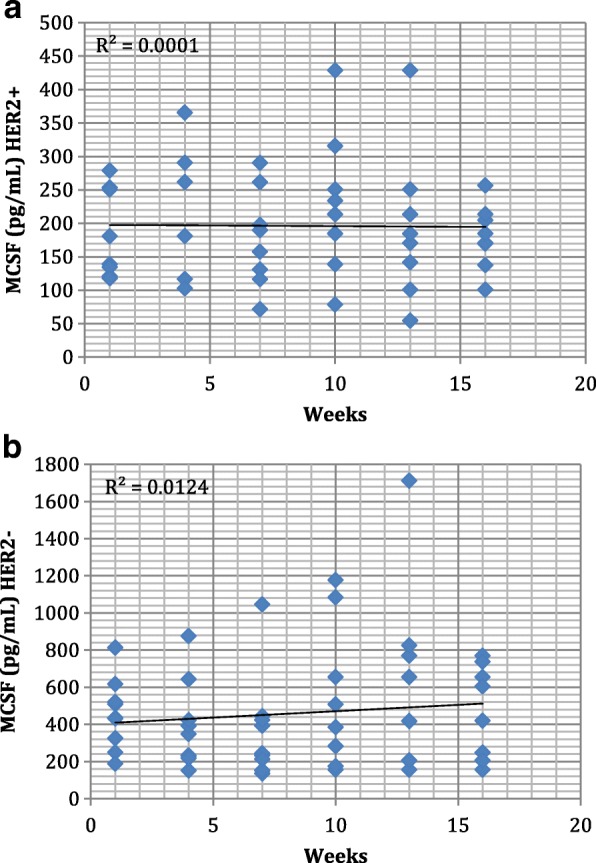
Table (**a**) Two-way scatter plots of MCSF serum levels in HER2+ patients (n = 96) and (**b**) HER2- patients (n = 108). Trend line along with R^2^ values is shown for each graph

Serum samples were submitted to VEGF and MCSF determination by using Elisa Kit (R&D Systems). VEGF basal levels were found to be higher in HER2- cohort (Fig 1b). In HER2+ cohort were observed a trend of stable levels of serum MCSF, ranging from (50 pg/mL to 450 pg/mL approximately) (Fig 2a) whereas VEGF levels were found slightly decreasing trend through TZM treatment (Fig 1a). In the HER2- cohort, we found an increasing trend for both VEGF and MCSF levels during treatment (Fig. 1 and 2).

## Discussion

One important scientific appeal of neoadjuvant chemotherapy is the possibility for analyzing the impact of systemic therapy in different BC subtypes, because tumor tissue biopsies can be obtained at different times during treatment, allowing for the development of biomarkers [8]. In this particular scenario, we were able to collect serum samples and analyze serum markers that might indicate the involvement of angiogenesis pathway in patients with known pathological response.

In the HER2+ cohorts under metronomic therapy with TZM, we observed higher pCR rates and downregulation of the proangiogenic factor VEGF. The ELISA kit used to determine VEGF levels is specific to detect soluble VEGF_165_ and VEGF_121_ among four isoforms (including bound VEGF_189_ and VEGF_206_). Thus the levels are indicative of the total circulating VEGF. Our findings corroborate previous observations indicating higher total soluble levels of VEGF in ductal carcinomas [24, 25]. TZM-treated group showed decreasing levels of VEGF during treatment which is in line with observations described by other groups. Since an activation of HER2−/neu has already been shown to increase the production of VEGF [15, 26], studies have suggested an inhibition of angiogenesis in BC by modulation of different (pro and anti) angiogenic factors due to TZM treatment [27]. Metronomic chemotherapy itself would lead to abrogation of newly formed tumor micro vessels and may synergistically impede its formation via downregulation of VEGF signaling promoted by TZM [28, 29]. Alternatively, the possible VEGF involvement in HER2+ patients under TZM treatment might be related to HER2+ biology per se, without interference of TZM activity; however, this hypothesis could be better understood with a randomized trial design. Our single arm trial design does not allow us to discriminate treatment from disease-related biology effects. Therefore, our work should be viewed as a hypothesis generating study only.

While most of our efforts to date have focused on its antiangiogenic properties, VEGF also has an immunologic role, inducing accumulation of immature dendritic cells, myeloid-derived suppressor cells, and regulatory T cells, and inhibiting the migration of T lymphocytes to tumor site [30]. The addition of metronomic chemotherapy to anti-HER2 treatment with TZM and Pertuzumab improve progression free survival in the elderly [31]. One possible mechanism may also involve immunosuppression through downregulation of regulatory T cells, enhancing antibody-dependent cell mediate cytotoxicity [31, 32]. In fact, abrogation of immunobiological responses has been seen in patients treated with anti-VEGF therapy [33]. Moreover, chemotherapy typically generates reactive oxygen species in targeted ECs, which can affect angiogenesis. Thus, BC angiogenesis is driven by multiple aspects that probably go well beyond the VEGF.

The anti-VEGF hypothesis in BC is difficult to reconcile with data from gastric cancer, for instance [34]. This may be explained in part because it is currently unknown which population would benefit from anti-VEGF therapy in BC. Studies examining anti-VEGF therapy in BC have been probed at both the genomic and proteomic levels [20]. However, to date no prospective trial using biomarkers approach has been published. Further evaluation of metronomic chemotherapy in combination with antiangiogenic agents could be an attractive strategy, although complicated by the fact that bevacizumab has failed clinical trials in the adjuvant setting in both HER2+ [35] and HER2- [36]. Bevacizumab program in this setting is now closed. Similarly, other antiangiogenic agents such as ramucirumab have failed in HER2-, in a BC randomized trial [37]. These results do not invalidate the strategy, but rather need to narrow targets to test hypothesis.

In our study, we did not observe down-regulation in VEGF or MCSF levels in HER2- patients under metronomic therapy. In a way, our data corroborate data from AVEREL study, a Phase III trial to evaluate bevacizumab in combination with TZM plus docetaxel for HER2+ advanced BC, that showed that a larger bevacizumab treatment effect was observed in patients with high baseline VEGF-A than in those with low VEGF-A [38]. The high local concentration of endothelial-cell survival factors such as VEGF and angiopoietin might cause further chemotherapy efficacy, being one possible mechanism of acquired resistance [39, 40]. The role of angiogenesis in chemo-resistance to TZM in advanced stages is one interesting topic for further investigation since validated biomarker to select potential responders does exist.

Here in we demonstrated potential evidence of angiogenic pathway involvement in the cancer biology of HER2+ patients under metronomic anti-EGF therapy. The sample size, the design of our work, in addition to the unexpected premature termination of the trial, prevents us from drawing firm conclusions. Further studies will be needed to validate our findings.

## Abbreviations

ASCO: American Society of Clinical Oncology
BC: Breast Cancer
CAP: College of American Pathologists
CR: Complete Response
EC: Endothelial Cell
ELISA: Enzyme-Linked Immunosorbent Assay
FGF 2: Fibroblast Growth Factor 2
HER2: Human Growth Factor Receptor 2
ICESP: Instituto do Cancer do Estado de São Paulo
LOCF: Last Observation Carried Forward
MCSF: Macrophage Colony Stimulating Factor
MMP2: Matrix Metalloproteinase 2
pCR: Complete Pathological Response
PR: Partial Response
SD: Stable Disease
TZM: Traztuzumab
VEGF: Vascular Endothelial Growth Factor
VEGFR: Vascular Endothelial Growth Factor Receptor
